# Methanol-Essential Growth of *Corynebacterium glutamicum*: Adaptive Laboratory Evolution Overcomes Limitation due to Methanethiol Assimilation Pathway

**DOI:** 10.3390/ijms21103617

**Published:** 2020-05-20

**Authors:** Guido Hennig, Carsten Haupka, Luciana F. Brito, Christian Rückert, Edern Cahoreau, Stéphanie Heux, Volker F. Wendisch

**Affiliations:** 1 Genetics of Prokaryotes, Faculty of Biology & CeBiTec, Universitätsstr. 25, Bielefeld University, 33615 Bielefeld, Germany; ghennig@cebitec.uni-bielefeld.de (G.H.); chaupka@cebitec.uni-bielefeld.de (C.H.); luciana.f.d.brito@ntnu.no (L.F.B.); 2 Department of Biotechnology and Food Science, Norwegian University of Science and Technology (NTNU), 7034 Trondheim, Norway; 3 Technology Platform Genomics, Center for Biotechnology (CeBiTec), Bielefeld University, Sequenz 1, 33615 Bielefeld, Germany; christian.rueckert@cebitec.uni-bielefeld.de; 4 Toulouse Biotechnology Institute (TBI), Université de Toulouse, CNRS, INRAE, INSA, 31077 Toulouse, France; cahoreau@insa-toulouse.fr (E.C.); heux@insa-toulouse.fr (S.H.)

**Keywords:** synthetic methylotrophy, methanol, ribulose monophosphate pathway, adaptive laboratory evolution, isotopic labeling, metabolic engineering

## Abstract

Methanol is a sustainable substrate for biotechnology. In addition to natural methylotrophs, metabolic engineering has gained attention for transfer of methylotrophy. Here, we engineered *Corynebacterium glutamicum* for methanol-dependent growth with a sugar co-substrate. Heterologous expression of genes for methanol dehydrogenase from *Bacillus methanolicus* and of ribulose monophosphate pathway genes for hexulose phosphate synthase and isomerase from *Bacillus subtilis* enabled methanol-dependent growth of mutants carrying one of two independent metabolic cut-offs, i.e., either lacking ribose-5-phosphate isomerase or ribulose-5-phosphate epimerase. Whole genome sequencing of strains selected by adaptive laboratory evolution (ALE) for faster methanol-dependent growth was performed. Subsequently, three mutations were identified that caused improved methanol-dependent growth by (1) increased plasmid copy numbers, (2) enhanced riboflavin supply and (3) reduced formation of the methionine-analogue O-methyl-homoserine in the methanethiol pathway. Our findings serve as a foundation for the engineering of *C. glutamicum* to unleash the full potential of methanol as a carbon source in biotechnological processes.

## 1. Introduction

Long-term transition from fossil fuels to renewable energy is inevitable and poses one of the major challenges for humanity in the 21st century. Methanol is a promising alternative renewable energy source with a price of 359 USD per metric ton (April 2020, Methanex, https://www.methanex.com/our-business/pricing) and the feasibility of a “methanol economy” has been proposed before [1]. Methanol can be synthesized from gasified biomass wastes or environmentally captured CO_2_—electrochemically or by hydrogenation. Therefore, pure H_2_ is required which can be gained from electrolysis of water, powered by solar, wind or thermal energy. [2]. Moreover, the conversion of CO_2_ to methanol by solar energy and the promising field of light-driven C_1_ chemistry in general are expected to replace conventional thermal catalytic processes in the chemical industry and it is estimated that 40–70 Mt of methanol will be produced from CO_2_ and H_2_ by 2050 [3].

Methylotrophic bacteria on plant surfaces function as a sink for volatile organic compounds such as methanol, the most abundant organic gas in the atmosphere besides methane and, thus, improve the overall air quality and minimize global warming [4]. Additionally, they are an important group of bioinoculants which are applied in sustainable agriculture to potentially substitute for chemical fertilizers in order to provide nitrogen and phosphorus to plants without depletion of soil quality [4].

Natural methylotrophy in bacteria has been studied extensively in the past decades to reveal three distinct pathways for biomass-related C_1_ utilization: the ribulose monophosphate pathway (RuMP) found e.g., in the Gram-positive, thermophilic *Bacillus methanolicus* [5], the serine cycle/ethyl-malonyl-CoA-pathway found e.g., in the Gram-negative, mesophilic *Methylobacterium extorquens* [6] and the ribulose bisphosphate pathway (RuBP) found e.g., in Gram-negative, autotrophic *Paracoccus denitrificans* [7]. The RuMP pathway was shown to be the most efficient pathway in terms of energy, as it yields one molecule of nicotinamide adenine dinucleotide (NADH) and adenosine triphosphate (ATP) for each pyruvate produced, whereas the serine cycle and RuBP require energy investments for carbon fixation and utilization [8,9].

Methanol is an attractive carbon source for biotechnological application and natural methylotrophs have successfully been used to produce valuable products, such as amino acids or fine chemicals [10]. Selected strains of *B. methanolicus* secreted up to 70 g/L of l-glutamate or 35 g/L of l-lysine in high-temperature fed-batch cultivations solely from methanol and the l-lysine/l-glutamate derived products cadaverine and gamma-aminobutyric acid have since been added to extend the product spectrum [11,12,13,14]. 

Although there have been recent advances in the development of tools for system engineering of *B. methanolicus* [15,16,17], the genetic toolbox for these methylotrophic bacteria is still limited and metabolic engineering to optimize carbon fluxes for increased product titers or yields remains a challenge to this day [18]. Thus, the demand to transfer methylotrophy to mesophilic bacteria with well-established engineering toolset has been high in recent years [19]. First attempts to engineer *Escherichia coli* and *Corynebacterium glutamicum* towards synthetic methylotrophy revealed functional operation of RuMP pathway enzymes but remained unsuccessful in establishing methanol utilization for biomass formation [20,21,22]. Recent approaches succeeded in re-routing the central carbon metabolism of non-methylotroph bacteria to create a methanol-dependency through metabolic cut-offs that required the co-utilization of methanol with gluconate or xylose through RuMP pathway reactions for partial complementation, which was subsequently enhanced through adaptive laboratory evolution (ALE) [23,24,25]. Moreover, a linear synthetic pathway named reductive glycine pathway was engineered in *E. coli* which enabled growth solely on C_1_ compounds [26].

Here, we engineered a methanol-dependency in *C. glutamicum* in two distinct approaches, deleting either the *rpi* gene, encoding ribose-5-phosphate isomerase or *rpe*, encoding ribulose-5-phosphate epimerase in the pentose-phosphate pathway. Hereby, we prevented the full metabolization of ribose, xylose or gluconate, forcing the co-utilization of methanol through RuMP pathway reactions for biomass formation. Methanol-dependent growth of the *rpe*-deletion strain was further improved until full complementation and 300% increased growth rate by ALE and mutations beneficial for methanol co-utilization were examined.

## 2. Results and Discussion

### 2.1. Methanol-Dependent Complementation of a Ribose 5-Phosphate Isomerase Mutant

**Figure 1:**
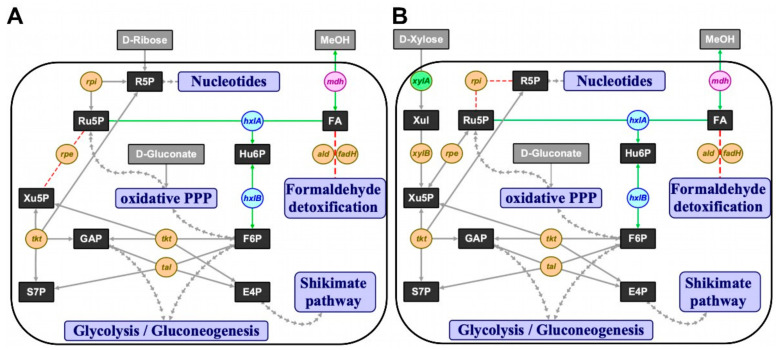
The ribulose monophosphate pathway (RuMP) implemented in *C. glutamicum*. (**A**) Δ*rpe* and (**B**) Δ*rpi* concepts for methanol-dependent complementation of two metabolic cut-offs of the pentose phosphate pathway in *C. glutamicum*, respectively. Substrates in grey boxes: MeOH, methanol; metabolites in black boxes: E4P, erythrose 4-phosphate; F6P, fructose 6-phosphate; FA, formaldehyde; GAP, glyceraldehyde 3-phosphate; Hu6P, hexulose 6-phosphate; R5P, ribose 5-phosphate; Ru5P, ribulose 5-phosphate; S7P, sedoheptulose 7-phosphate; Xul, xylulose; Xu5P, xylulose 5-phosphate; interconnected pathways, violet boxes: PPP, pentose phosphate pathway; native or homologous overexpression of genes in orange circles: *rpe*, ribulose 5-phosphate epimerase; *rpi*, ribose 5-phosphate isomerase; *tal*, transaldolase; *tkt*, transketolase; *xylA*, xylose isomerase; *xylB*, xylulokinase; heterologous overexpression of *xylA* gene (xylose isomerase) from *X. campestris* in green circle; heterologous overexpression of RuMP pathway genes from *B. subtilis* in blue circles: *hxlA*, 3-hexulose 6-phosphate synthase; *hxlB*, 6-phospho 3-hexulose isomerase; heterologous overexpression of *mdh* gene (methanol dehydrogenase) from *B. methanolicus* in pink circle; red arrows, knocked out reactions; green arrows, complementing reactions.

Since methanol is a convenient alternative carbon source to sugar substrates that are commonly used in biotechnological processes and metabolic engineering of native methylotrophs is still limited, recent efforts focused on establishing synthetic methylotrophy bacteria to produce high-value chemicals from methanol [20,23,24,25]. Since methanol is more reduced than glucose, it supports production of reduced compounds. Although omics data of native methylotrophs have been generated in the past decade [6,15,16,27], natural methanol utilization has not yet been fully understood, which makes the transfer of methylotrophy to non-methylotrophs a demanding challenge [5,19]. Here, we developed methanol-dependent *C. glutamicum* strains through *rpe* and *rpi* deletions that functioned as metabolic cut-offs to force methanol-utilization for biomass formation (Figure 1A,B).

**Figure 2:**
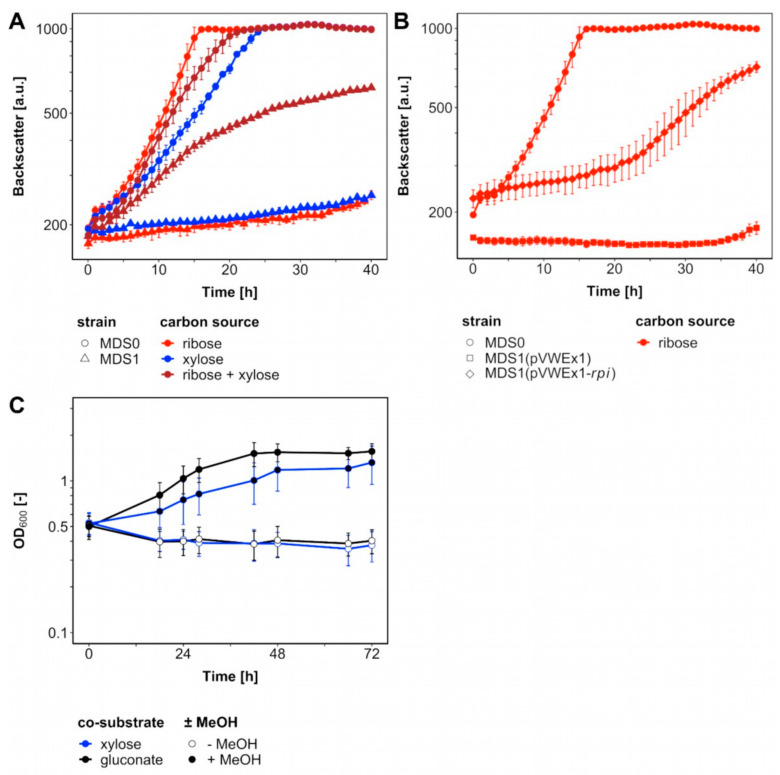
Loss and gain of Rpi Function. *C. glutamicum* strains cultivated in BioLector with 1 mL CGXII medium including carbon sources and 1 mM isopropyl β-D-thiogalactopyranoside (IPTG) at 30 °C. (**A**) Mutants Δ*ald* Δ*fadH* (MDS0) (circles) and Δ*ald* Δ*fadH* Δ*rpi* (pEKEx3-*xylAB)* (MDS1) (triangles) comprising plasmid pEKEx3-*xylAB* were supplemented with 20 mM ribose (red), xylose (blue) and both (brown). (**B**) MDS0 (circles), MDS1(pVWEx1) (rectangles) and MDS1(pVWEx1-*rpi*) (diamonds) were supplemented with 20 mM ribose (red). (**C**) MDS1(pVWEx1-*mdh*-*hxlAB*) cultivated in 100 mL shakings flasks with 10 mL CGXII medium including carbon sources, 0.1 g L^−1^ yeast extract and 1 mM IPTG at 30 °C. Supplementation with either 20 mM xylose (blue) or gluconate (black) with and without 500 mM methanol (full, empty). Error bars indicate standard deviations of biological triplicates.

A metabolic cut-off to isolate ribose 5-phosphate-dependent anabolism from the rest of the cellular metabolism was constructed by deletion of ribose 5-phosphate isomerase gene *rpi*. Growth of this pentose phosphate pathway mutant with xylose, which required transformation with the plasmid pEKEx3-*xylAB*, was expected only upon supplementation with ribose (Figure 1B). Indeed, strain Δ*ald* Δ*fadH* Δ*rpi* (pEKEx3-*xylAB)* (MDS1) grew poorly on lysogeny broth (LB) or brain heart infusion (BHI) plates, whereas BHI plates supplemented with 20 mM ribose, 20 mM xylose and 1 mM isopropyl β-D-thiogalactopyranoside (IPTG) supported efficient growth (data not shown). In minimal media, strain MDS1 grew with a rate of 0.05 h^−1^ with a mixture of ribose and xylose, but it did neither grow with xylose nor ribose as sole carbon source (Figure 2A). The parental strain MDS0(pEKEx3-*xylAB*), used as positive control, grew with growth rates of 0.15 h^−1^ with ribose, 0.08 h^−1^ with xylose and 0.10 h^−1^ with the mixture of xylose and ribose (Figure 2A). Next, we genetically complemented *C. glutamicum* strain MDS1 by transformation with pVWEx1-*rpi*. After lag phase, the strain MDS1(pVWEx1-*rpi*) grew on ribose as sole carbon source, while the empty vector carrying the control strain MDS1(pVWEx1) did not (Figure 2B). Thus, loss of function of *rpi* caused the observed growth phenotype.

In order to test if a functional RuMP cycle for formaldehyde assimilation can circumvent feeding ribose to the *rpi* deletion mutant (Figure 1B), strain MDS1(pVWEx1-*mdh*-*hxlAB*) for expression of the genes encoding methanol dehydrogenase from *B. methanolicus* and 3-hexulose 6-phosphate synthase HxlA and hexulose 6-phosphate isomerase HxlB from *B. subtilis* was constructed and evaluated for methanol-dependent growth (Figure 2C). No growth of this strain was observed with xylose as sole carbon source, however, upon addition of methanol the strain grew with a growth rate 0.03 h^−1^ from an OD_600_ of 0.52 ± 0.07 to 1.39 ± 0.37. As an independent test, growth with gluconate as sole carbon source with/without addition of methanol was tested since ribulose 5-phosphate is generated during gluconate catabolism. Indeed, strain MDS1(pVWEx1-*mdh*-*hxlAB*) grew with a growth rate of 0.04 h^−1^ from an initial OD600 of 0.50 ± 0.07 to 1.63 ± 0.10. Although no ALE was conducted with strain MDS1, the specific growth rates with both co-substrates were on par with, but not faster than previously reported growth rates for methanol-dependent growth of evolved *C. glutamicum* strain MX-11 with xylose (0.03 h^−1^) [25].

To exclude that *rpi* deletion mutants can only be selected for if compensatory mutations occurred elsewhere in the genome, we re-sequenced the genome of strains MDS0(pEKEx3-*xylAB*)(pVWEx1-*mdh*-*hxlAB*) and MDS1(pVWEx1-*mdh*-*hxlAB*) that only differ by the presence/absence of *rpi*. For strain MDS0(pEKEx3-*xylAB*)(pVWEx1-*mdh*-*hxlAB*), 79.25%, 14.23% and 7.17% of the processed reads could be mapped to the genome, plasmid pEKEx3-*xylAB* and plasmid pVWEx1-*mdh*-*hxlAB*, respectively. For the *rpi* deletion mutant MDS1(pVWEx1-*mdh*-*hxlAB*), 79.81%, 13.43% and 6.33% of the processed reads were mapped to the genome, plasmid pEKEx3-*xylAB* and plasmid pVWEx1-*mdh*-*hxlAB*, respectively. As expected, reads mapping to the deleted genes *ald* (cg3096) and *fadH* (cg0387) were not found for both strains. As compared to the reference genome sequence, in both strains *psp1* (cg2069) coding for a putative secreted protein of the prophage CGP3 was only partly covered and seven non-silent single-nucleotide polymorphisms (SNPs) were found (Table 1). Three occurred in the remainders of the deleted genes *ald* and *fadH* which were left to ensure in frame-deletions and prevent polar effects. The gene functions of *loci* cg0822 and cg1245 are yet unknown and additional SNPs were found in *wzz* and *hrtA* (cg0414, cg2204) encoding for proteins involved in cell surface polysaccharide biosynthesis, chain length determination and an ABC-type transport system and ATPase component [28]. As expected, none of the SNPs matched the ones generated after ALE of a *C. glutamicum* strain with a similar background [25]. Importantly, while sequenced reads were mapped to the *rpi locus* (cg2658) for strain MDS0(pEKEx3-*xylAB*)(pVWEx1-*mdh*-*hxlAB*), with a mean feature coverage of 12, no reads were mapped to this gene in strain MDS1(pVWEx1-*mdh*-*hxlAB*). Thus, the presence/absence of *rpi* was the only difference between the strains.

**Figure 3:**
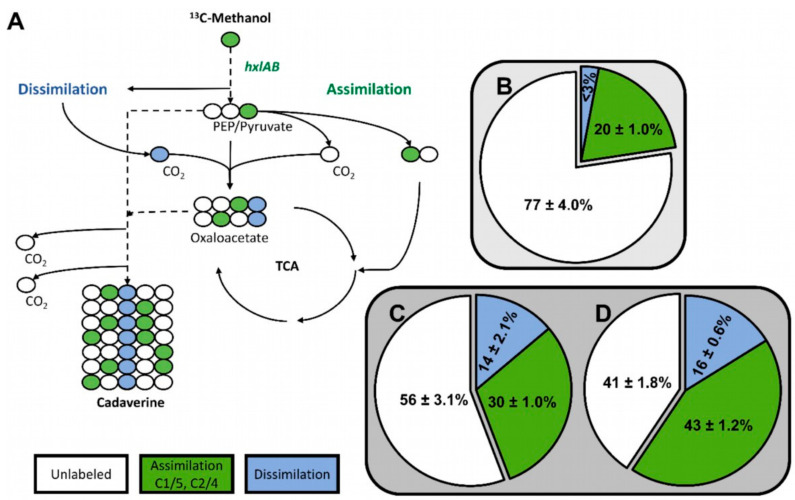
Labeling of cadaverine from ^13^C-methanol by *C. glutamicum* strains MDS1 and MDS2T8. (**A**) Possible ^13^C-isotopologue distributions in the central carbon pathway. (**B**) Cadaverine was produced by the methanol-independent strain *C. glutamicum* MDS0 (pEKEx3-*mdh*-*hxlAB*) (pVWEx1-*lysC*^fbr^-*ldcC*) [20] (**C**) MDS1 (pVWEx1-*mdh*-*hxlAB*) (pEC-XT99A-*lysC^fbr^*-*ldcC*) and (**D**) MDS2T8 (pEC-XT99A-*lysC^fbr^*-*ldcC*), respectively in shake flasks containing M9 minimal medium with 20 mM gluconate, 0.5 g/L yeast extract and 500 mM ^13^C-methanol for 72 h. The pie charts show mean ^13^C enrichment in cadaverine determined at carbon positions C1/C5 and C2/C4 for assimilation of carbon via RuMP pathway reactions (green) and C3 for assimilation of ^13^CO_2_ that has arisen from dissimilation of ^13^C-methanol (blue).

To determine if methanol was assimilated via the RuMP pathway in the *C. glutamicum* strain MDS1(pVWEx1-*mdh*-*hxlAB*), the fate of ^13^C-methanol was followed in the non-native product cadaverine. As reported previously, cadaverine production was enabled by overexpression of endogenous, feedback resistant aspartokinase (*lysC*^T311I^) and heterologous expression of lysine decarboxylase (*ldcC*) from *E. coli* (using plasmid pEC-XT99A-*lysC*^fbr^-*ldcC* [20]. Non-labeled gluconate was chosen as secondary substrate to ^13^C-methanol to provide additional NADPH, benefitting production of cadaverine precursor L-lysine [20]. In the chosen ^13^C-labeling condition (20 mM gluconate and 500 mM ^13^C-methanol), labeling at the C1/C5 or C2/C4 positions of the cadaverine molecule indicates utilization of methanol via RuMP pathway reactions, whereas C3 labeled cadaverine results from anaplerotic carboxylation reactions with ^13^CO_2_ that arose from oxidation of ^13^C-methanol to ^13^CO_2_ (Figure 3A) [20]. The ^13^C-labeling pattern in 1.5 mM cadaverine that were produced after 48 h by MDS1(pVWEx1-*mdh*-*hxlAB*)(pEC-XT99A-*lysC*^fbr^-*ldcC*) besides production of 1.5 mM L-lysine indicated that 30 ± 1.0% of the cadaverine molecules originated from functional operation of the RuMP pathway, 14 ± 2.1% originated from ^13^CO_2_ and 56 ± 3.1% were unlabeled (Figure 3C). This indicates a 50% increased RuMP functionality over strain MDS0(pVWEx1-*mdh*-*hxlAB*)(pEC-XT99A-*lysC*^fbr^-*ldcC*) of which 20 ± 1.0% of the produced cadaverine was labeled at the C1/C5 or C2/C4 positions (Figure 3B) [20] and is comparable to 24% methanol assimilation into hexose 6-phosphate achieved by engineered and evolved *E. coli* strains [24].

### 2.2. Adaptive Laboratory Evolution for Accelerated Methanol-Dependent Complementation of a Ribulose 5-Phosphate Epimerase Mutant

**Figure 4:**
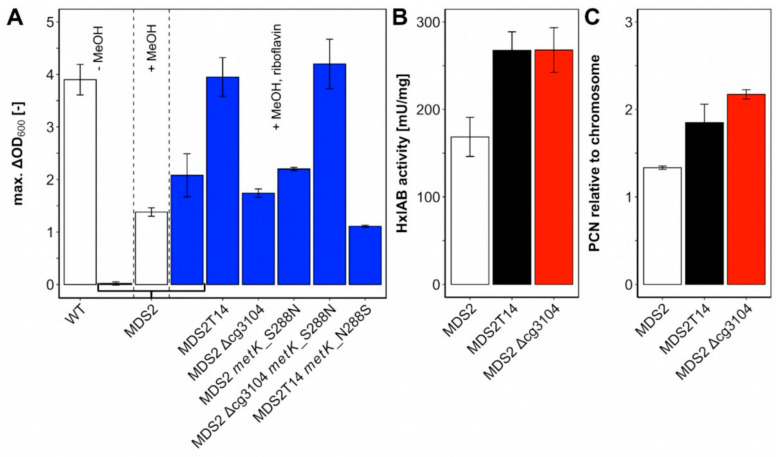
Biomass formation with methanol as co-substrate (**A**) and relative copy numbers of plasmid pEKEx3-*mdh*-*hxlAB* (**B**) and HxlAB activities (**C**) in strain *C. glutamicum* MDS2 and its derivatives. (**A**) *C. glutamicum* wildtype (WT) and MDS2 were cultivated in CGXII medium with 20 mM ribose for 48 h and the maximal ΔOD was determined. *C. glutamicum* Δ*ald* Δ*fadH* Δ*rpe* (pEKEx3-*mdh*-*hxlAB*) (= MDS2), and derivatives were cultivated in CGXII medium containing 20 mM ribose and 500 mM methanol for 48 h to determine the maximum ΔOD and investigate the influence of mutations found in strain MDS2T14, obtained by ALE. Blue bars indicate addition of 20 µM riboflavin. (**B**) Quantitative PCR (qPCR) was performed on isolated genomic and plasmid DNA from overnight cultures of MDS2, MDS2T14 and MDS2 Δcg3104. Relative plasmid copy numbers (PCN) were calculated using serial dilutions from 100 to 0.1 ng/μL as standard curve for the chromosomal (*gntK*) and plasmid (*oriV_Cg_*) targets. Error bars depict standard deviations of technical triplicates. (**C**) Coupled HxlAB enzyme assays were performed with crude extracts of MDS2, MDS2T14 and MDS2 Δcg3104.

To reduce the number of plasmids to one, we first constructed a new strain containing only one plasmid. Instead of *rpi*, the ribulose 5-phosphate epimerase gene *rpe* was deleted in MDS0 to isolate ribose-5-phosphate dependent anabolism from the rest of cellular metabolism. As a result, only one plasmid (i.e., pEKEx3-*mdh*-*hxlAB*) was required since in this metabolic setting *C. glutamicum* naturally catabolizes ribose. Methanol-dependency of the new *C. glutamicum* strain MDS2(pEKEx3-*mdh*-*hxlAB*) was examined in minimal medium with 20 mM ribose as co-substrate. No growth with ribose as sole carbon source was observed for 72 h. By contrast, with methanol plus ribose, strain MDS2(pEKEx3-*mdh*-*hxlAB*) formed biomass (ΔOD of 1.32 ± 0.03) with specific growth rate of 0.03 ± 0.01 h^−1^ (Figure 4A). Thus, methanol dependent growth of two-vector strain MDS1(pVWEx1-*mdh-hxlAB*) with methanol plus xylose and one-vector strain MDS2(pEKEx3-*mdh*-*hxlAB*) with methanol plus ribose were comparable.

*C. glutamicum* strain MDS2(pEKEx3-*mdh*-*hxlAB*) was subjected to ALE by iterative transfers of cells grown in minimal medium with ribose and methanol. After the first eight transfers, strain MDS2T8 was conserved for whole-genome sequencing and used to determine to what extent ^13^C-methanol is assimilated via the RuMP pathway.

Carbon labeling of cadaverine from ^13^C-methanol was investigated to determine if methanol is assimilated via the RuMP pathway in *C. glutamicum* strain MDS2T8 transformed with pEC-XT99A-*lysC*^fbr^-*ldcC*. The ^13^C-labelling pattern of 1.6 mM cadaverine besides 1.6 mM L-lysine produced by MDS2T8(pEC-XT99A-*lysC*^fbr^-*ldcC*) indicated that 43% of the cadaverine molecules originated from operation of the RuMP pathway, 16% originated from ^13^CO_2_ and 41% were unlabeled (Figure 3D). Thus, the strain selected by ALE showed a higher proportion of cadaverine molecules originating from a functional RuMP pathway as compared to the non-evolved strain MDS1(pEKEx3-*xylAB*)(pVWEx1-*mdh*-*hxlAB*)(pEC-XT99A-*lysC*^fbr^-*ldcC*), whereas the dissimilatory pathway activity of both strains was similar (16% vs. 14%).

Furthermore, transfers of *C. glutamicum* strain MDS2T8 were performed from minimal medium to minimal medium with 0.5 g/L of yeast extract. After six further transfers, *C. glutamicum* strain MDS2T14 was selected as it reached a final ΔOD of 3.95 ± 0.37 in minimal medium with ribose and methanol with a specific growth rate of 0.10 ± 0.01 h^−1^, which is comparable to the wild type (WT) grown with 20 mM ribose (ΔOD of 3.90 ± 0.29) (Figure 4A). Therefore, MDS2T14 was 3-times as fast as strain *C. glutamicum* MX-11 which was cultivated with xylose as co-substrate [25] and exceeded the growth rate of evolved methanol-essential growth with *E. coli* co-utilizing gluconate (0.081 ± 0.002 h^−1^) [24]. Notably, strain MDS2T14 did not require yeast extract for methanol-dependent growth. Taken together, ALE allowed the selection of a mutant strain of MDS2 named MDS2T14 that showed full methanol-dependent complementation of the *rpe* deletion.

### 2.3. Genome Sequencing of ALE Strains Revealed Candidate Mutations that May Accelerate Methanol-Dependent Biomass Formation

**Figure 5:**
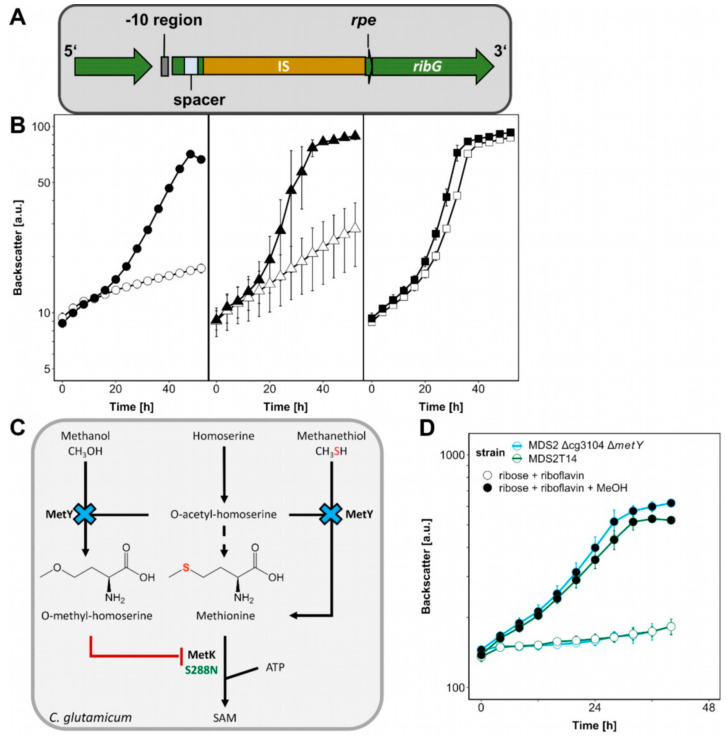
The influence of ALE mutations on methanol-dependent growth. (**A**) The insertion at the *rpe locus* in MDS2T14 is depicted. (**B**) *C. glutamicum* MDS2 (circles), MDS2T8 (triangles), MDS2T14 (squares) were cultivated using the BioLector with 1 mL filling volume of CGXII minimal medium with 20 mM glucose as sole carbon source (open symbols) or supplemented with 20 μM riboflavin (filled symbols). Backscatter was detected in BioLector with a gain of 20. Error bars depict standard deviations from biological triplicates. (**C**) The effect of Δ*metY* and *metK*^S288N^ on methanol-essential growth and S-adenosylmethionine (SAM) synthesis is shown. (**D**) *C. glutamicum* strain MDS2 Δcg3104 Δ*metY* was cultivated using the BioLector with 1 mL filling volume of CGXII minimal medium with 20 mM ribose and 20 μM riboflavin (empty symbols) and additional 500 mM methanol (filled symbols). Backscatter was detected in BioLector with a gain of 20. Error bars depict standard deviations from biological triplicates.

The genome sequences of ALE strains MDS2T8 and MDS2T14 were determined and compared to that of the parental strain MDS2 to identify candidate mutations that may be causal for accelerated methanol-dependent growth. As compared to the parental *C. glutamicum* strain MDS2, strain MDS2T8 possessed three mutations: non-silent SNPs in the genes *metK* (S-adenosylmethionine synthetase) and *res* (site-specific recombinase) resulting in amino acid exchanges S288N and R91H, respectively, and a 15 bp deletion disrupting the coding sequence (CDS) of an uncharacterized gene (cg3104) which is annotated as ATPase involved in DNA repair. The latter shares structural motifs typically found in proteins with DNA exo- and endonuclease activity, such as the well-characterized SbcCD protein in *E. coli* [29] that was revealed to be capable of cleaving secondary hairpin structures during plasmid replication [30]. The unique mutation found in strain MDS2T14 was a 1.4 kb transposon insertion near the deleted *rpe* gene which shares a leaderless promoter with the riboflavin synthesis genes *ribG* and *ribC*, encoding putative bifunctional riboflavin specific deaminase/reductase and putative riboflavin synthase, respectively (Figure 5A) [31].

### 2.4. Identification of Mutations Causal for Methanol-Essential Growth

First, we analyzed the effect that deletion of *rpe* has on riboflavin biosynthesis. *C. glutamicum* MDS2(pEKEx3-*mdh*-*hxlAB*) and the evolved strains MDS2T8 and MDS2T14 were cultivated on glucose as sole carbon source with or without riboflavin supplementation to find out whether the transposon integration in MDS2T14 influenced growth without riboflavin. Growth of strains MDS2 and MDS2T8 on glucose minimal medium could be enhanced by riboflavin addition (Figure 5B) as it was previously reported in studies investigating riboflavin transport in a riboflavin deficient *C. glutamicum* strain [32]. By contrast, without added riboflavin strain MDS2T14 grew as fast as or faster than MDS2 and MDS2T8 with added riboflavin (Figure 5B). Thus, the growth advantage of strain MDS2T14 is riboflavin-dependent and likely due to the transposon integration which provided an additional potential -10 promoter region (TATTT) upstream of *ribG* and an alternative ribosome binding motif (AAGGG) 7 bp upstream of the *ribG* start-codon [33,34].

Deletion of cg3104 in parental strain MDS2 did not increase biomass formation (ΔOD: 1.74 ± 0.08) as compared to MDS2 (ΔOD: 2.08 ± 0.41) (Figure 4A). Based on its supposed function as putative ATPase involved in DNA repair, the plasmid copy number of plasmid pEKEx3-*mdh*-*hxlAB* was determined by qPCR (Figure 4C). Strains MDS2T14 and MDS2 Δcg3104 showed approximately 40–60% increased relative plasmid copy number (1.85 ± 0.21 and 2.17 ± 0.05, respectively) as compared to that of the parental strain MDS2 (1.33 ± 0.02). Because of the elevated copy numbers, higher enzyme activities of the heterologously expressed RuMP genes were expected. A coupled assay of HxlA and HxlB revealed 60% higher enzyme activities (*p* < 0.05) of MDS2T14 (268 ± 21 mU/mg) and MDS2 Δcg3104 (268 ± 26 mU/mg) compared to MDS2 (169 ± 22 mU/mg) (Figure 4B), which is beneficial for formaldehyde fixation. Improved channeling of formaldehyde into central metabolites has been shown to shift the equilibrium of Mdh towards the methanol-oxidation reaction in *E. coli,* for an overall increased utilization of methanol [35]. Efficient methanol-oxidation and formaldehyde channeling has been argued to be one of the major bottlenecks for synthetic methylotrophy [24,36], which is further reinforced by our finding that increased HxlAB activity is an essential factor for improved growth of strain MDS2T14.

Another key factor for improved methanol-dependent growth of MDS2T14 is an amino acid exchange from serine to asparagine found at position 288 in MetK (Table 1) which is next to the methionine binding K289. MetK catalyzes the formation of S-adenosylmethionine (SAM) from methionine and ATP (Figure 5C) [37]. Both *E. coli* and *C. glutamicum* only harbor one copy of the essential *metK* gene [38]. When combining deletion of cg3104 with introduction of MetK amino acid exchange S288N, growth with ribose and methanol was improved as strain *C. glutamicum* MDS2 Δcg3104 *metK*_S288N grew to a ΔOD of 4.20 ± 0.47, reaching comparable levels of biomass formation as the evolved strain MDS2T14 (3.95 ± 0.37) and *C. glutamicum* WT with 20 mM ribose (3.90 ± 0.29) (Figure 4A). Introduction of the MetK amino acid exchange S288N alone had no positive effect on biomass formation (ΔOD: 2.20 ± 0.03). However, reverting the amino acid exchange in MetK in the evolved strain MDS2T14 from S288N to N288S, i.e., to the WT sequence, reduced growth on ribose and methanol considerably (ΔOD of 1.11 ± 0.02) (Figure 4A). Taken together, the MetK amino acid exchange S288N combined with deletion of cg3104 and supplementation with riboflavin was sufficient to explain improved growth of the evolved strain MDS2T14 in minimal medium with ribose and methanol. 

The antimicrobial methionine analogue O-methyl-homoserine [39] was shown to be a naturally occurring side-product of O-acetyl-homoserine sulfhydrolase (MetY) of *Corynebacterium acetophilum* in the presence of homoserine and methanol since the latter is an analogue of methanethiol, the precursor of methionine (Figure 5C) [40]. The accumulation of growth-inhibiting O-methyl-homoserine in presence of methanol has been argued to be connected to methanol tolerance before, when MetY was found to be mutated leading to amino acid substitution A165T in a methanol tolerant *C. glutamicum* strain [41]. Furthermore, *metY* was among the mutated genes of strain *C. glutamicum* MX-11 that exhibited improved methanol-dependent growth with xylose [25]. During the writing process of this manuscript, a follow-up study from the same group appeared, further elucidating this mutation (G419D) [42]. It was shown to increase the tolerance for methanol, which was confirmed by knock-down and knock-out experiments. Here, we observed elevated methanol-dependent growth with ribose and riboflavin after *metY* deletion in strain MDS2 Δcg3104 (Figure 5D), which was similar to strain MDS2 Δcg3104 *metK*_S288N, indicating that both encoded enzymes, MetK and MetY, presumably act on each other when methanol is present and significantly improve methanol-dependent biomass formation of *C. glutamicum*.

## 3. Materials and Methods 

### 3.1. Microorganisms and Cultivation Conditions

*E. coli* DH5α strain was used as a host for cloning [43], S17-1 for transconjugation [44]. Both strains were grown in lysogeny broth (LB) at 37 °C and supplemented with antibiotics (50 μg mL^−1^ kanamycin, 100 μg mL^−1^ spectinomycin, 10 μg mL^−1^ tetracycline) when appropriate. *C. glutamicum* ATCC13032 and derived mutants were cultivated in brain heart infusion (BHI), or CGXII minimal medium [45] supplemented with 25 μg mL^−1^ kanamycin, 100 μg mL^−1^ spectinomycin, 5 μg mL^−1^ tetracycline, and 1 mM IPTG when appropriate. All strains and plasmids are listed in Table 2 and Table 3.

For growth experiments, *C. glutamicum* overnight cultures in 50 mL BHI were harvested, washed twice in CGXII medium, inoculated to an OD_600_ of 0.5 in 10 mL CgXII and supplemented with different carbon source combinations of methanol (500 mM) with gluconate, ribose or xylose (20 mM each) and 20 µM riboflavin for ALE experiments at 30 °C and 120 rpm. Growth was monitored in 100 mL shake flasks, by determination of the OD_600_ with V-1200 Spectrophotometer (VWR, Radnor, PA, USA) and with a 48-well flower plate in the Biolector microfermentation system (m2p-labs, Baesweiler, Germany), respectively.

### 3.2. ALE Experiments

ALE of *C. glutamicum* strain MDS2 (methanol-dependent strain) was carried out in two phases: In phase 1 [MDS2 – MDS2T8] triplicates were cultivated in CGXII minimal medium with 20 mM ribose and 500 mM methanol with intermediate inoculations of LB overnight cultures and vice versa from the highest grown culture. In phase 2 [MDS2T8-MDS2T14], transfers to LB were omitted and minimal medium was supplemented with 0.5 g/L yeast extract, 20 mM ribose and 500 mM methanol.

### 3.3. Molecular Biology Methods

Genomic DNA of *C. glutamicum* was isolated as described previously [51]. Classical methods including plasmid isolation, molecular cloning and transformation of *E. coli* by heat shock and of *C. glutamicum* by electroporation were performed as described before [45]. DNA sequences were amplified with the ALLin HiFi DNA Polymerase (HighQu, Kraichtal, Germany) using plasmid or genomic DNA as template. The oligonucleotides used in this study are listed in Table S1. The gene *rpi* was amplified from *C. glutamicum* and assembled into PstI- and BamHI-digested pVWEx1 by Gibson Assembly using the respective primers. Likewise, the genes *mdh*, *hxlA* and *hxlB* were amplified as a single operon from the plasmid pEKEx3-*mdh*-*hxlAB* [20] and cloned into pVWEx1 and pEC-XT99A linearized with BamHI. The constructed plasmids and empty vectors were transferred into *C. glutamicum* by transformation. For deletion, plasmid pK19*mobsacB* [44] digested with EcoRI and BamHI was assembled with amplified DNA fragments flanking the genes *rpe* and cg3104 by Gibson Assembly and transferred into *E. coli* S17-1 to follow a protocol for gene deletion routinely applied [45].

### 3.4. Coupled in vitro Activity of HxlA and HxlB

*C. glutamicum* cells were grown overnight in BHI medium containing 1 mM IPTG and appropriate antibiotics, harvested by centrifugation (5 min, 4000 rpm), washed in 50 mM phosphate buffer and subsequently disrupted by sonication with an amplitude of 50% and a duty circle of 0.5 for 9 min. Afterwards, total protein concentrations were determined by the Bradford method with bovine serum albumin as standard. Activities of HxlA and HxlB in crude extracts were determined in a coupled assay, derived from a previously described method [20]. A total volume of 1 mL was used for measurements, containing 25 mM phosphate buffer pH 7.4, 5 mM MgCl_2_, 5 mM ribose-5-phosphate, 0.5 mM NAD^+^, 2 U phosphoriboisomerase from spinach (Sigma), 2 U phosphoglucoisomerase from yeast (Sigma), 2 U Glucose-6-P dehydrogenase from *Leuconostoc mesenteroides* (Sigma) and 50 μL crude extract. An addition of 5 mM formaldehyde started the reaction and NADH formation was continuously measured at 340 nm and 30 °C for 6 min.

### 3.5. Whole-Genome Sequencing

Whole-genome sequencing was performed with isolated genomic DNA from *C. glutamicum* strains (PRJNA603493). DNA library preparation, trimming and mapping of the reads and visualization was performed as described previously [52]. For SNP detection in all CDS’s of *C. glutamicum*, the built-in tool from ReadXplorer was used. Minimal scores for base quality, average base quality and average mapping quality were set to 20 and reads with a coverage between 5 and 10 were kept if the corresponding frequency was 100%. If the coverage was above 10, the minimum frequency was 90% instead. Additionally, genomic DNA of *C. glutamicum* Δ*ald* Δ*fadH* Δ*rpe* strains MDS2, MDS2T8 and MDS2T14 was sequenced using Nanopore MinION sequencing technology (Oxford Nanopore Technologies Oxford, UK) as described previously [53]. After assembly, 11 contigs for MDS2, 6 contigs for MDS2T8 and 2 contigs for MDS2T14 were revealed. Assembled genomes of MDS2T8 and MDS2T14 were aligned with the MDS2 genome sequence using SnapGene software v.4.3 (GSL Biotech, Chicago, USA) to visualize SNPs and insertions or deletions. Variant calling was performed manually to identify SNPs in the MDS2T8 and MDS2T14 genomes, after alignment to the MDS2 parental strain genome reference.

### 3.6. Quantitative PCR

Quantitative PCR (qPCR) was performed in 96-well plates, sealed with transparent adhesive cover, in a CFX Connect^TM^ Real-Time PCR Detection System (Biorad, Hercules, USA). A total volume of 20 µL contained 10 µL SensiFAST^TM^ SYBR No-ROX Kit from Bioline (Heidelberg, Germany), 100 nmol/L forward/reverse primer and 4 µL of diluted template DNA. Genomic template DNA, also containing plasmid DNA, was isolated as described before. Fragments of approximately 250 bp were amplified, targeting *gntK* encoding gluconate kinase on the chromosome and *oriV_Cg_* on the pEKEx3 plasmid. Serial dilutions of DNA from 100 to 0.1 ng/µL were used to construct relative standard curves for chromosomal and plasmid targets. Plasmid and chromosome-specific amplicons were additionally detected in separate reactions and technical triplicates at 10 ng/µL to calculate plasmid copy numbers.

### 3.7. Quantification of ^13^C-Enrichment

Cultures of *C. glutamicum* MDS1 and MDS2T8 (pEC-XT99A-*lysC*^fbr^-*ldcC*), grown overnight in BHI medium, containing 1 mM IPTG and antibiotics were used to inoculate modified M9 medium with 20 mM gluconate and 500 mM ^13^C-methanol to a starting OD_600_ of 0.5. Modified M9 medium contained 7.5 g/L Na_2_HPO_4_, 3 g/L KH_2_PO_4_, 0.5 g/L NaCl, 0.25 g/L MgSO_4_ 7 H_2_O, 0.003 g/L CaCl_2_, 0.5 g/L NH_4_Cl and the trace elements as described for CGXII medium [45]. Samples of 1 mL were taken after 24, 48 and 72 h of cultivation and subsequently centrifuged for 10 min at 10,000 rpm. Supernatants were frozen at −20 °C for analysis of isotopologues as previously described [20].

## 4. Conclusions

By engineering two alternative metabolic cut-offs in the central carbon metabolism of *C. glutamicum* to reinforce methanol utilization through RuMP pathway reactions, we achieved methanol-dependent biomass formation on three different co-substrates (ribose, xylose and gluconate). Incorporation of methanol-carbon and functionality of the introduced RuMP pathway was confirmed by following the fate of ^13^C-methanol in cadaverine. Growth rates and methanol-dependent biomass formation were improved through ALE. Analysis of mutations identified by genome sequencing of evolved strains revealed three mutations causing the observed metabolic changes concerning improved methanol utilization through an increased plasmid copy number, resistance against methionine-analogue toxicity due to modified *metK* sequence, which was also achieved by *metY* deletion and enhanced riboflavin supply because of alternative promoter and RBS motifs. The findings reported here will serve as a basis for future engineering approaches towards synthetic methylotrophy in *C. glutamicum* and lead to a better understanding of necessary requirements for synthetic methylotrophy in general to unleash the full potential of methanol as biotechnological carbon source for the sustainable production of valuable chemical compounds in the future.

## Figures and Tables

**Table 1:** Single-nucleotide polymorphisms (SNPs) determined by whole-genome sequencing. Non-silent SNPs and mutations found in the strains ∆*ald* ∆*fadH* and ∆*ald* ∆*fadH* ∆*rpi*, as compared to the wildtype *C. glutamicum* ATCC13032, or strains MDS2T8 and MDS2T14 in comparison to their parental strain MDS2, including the corresponding *loci*, gene names, products and the resulting amino acid substitutions.

Locus	Gene Name	Strain	Gene Product	Mutation/Effect
cg0387	*fadH*	MDS0	mycothiol-dependent formaldehyde dehydrogenase	V9F
cg0414	*wzz*	MDS0	cell surface polysaccharide biosynthesis/chain length determinant protein	E363D
cg0822	-	MDS0	unknown	V23A
cg1245	-	MDS1	putative membrane protein	L328S
cg2204	*hrtA*	MDS1	ABC-type transport system, involved in lipoprotein release, ATPase component	D67H
cg3096	*ald*	MDS1	acetaldehyde dehydrogenase	T2S
cg3096	*ald*	MDS1	acetaldehyde dehydrogenase	L494M
cg3104	-	MDS2T8	putative ATPase involved in DNA repair	15 bp deletion within coding sequence (CDS)
cg1806	*metK*	MDS2T8	S-adenosylmethionine synthetase	S288N
cg1929	*res*	MDS2T8	Site-specific recombinase	R91H
cg1801	*rpe*	MDS2T14	Ribulose-5-phosphate epimerase	Insertion of a 1.4 kb transposon

**Table 2:** Bacterial strains used in this study.

Strain	Relevant Characteristics	Reference
*E. coli*		
DH5α	∆*lac*U169 (φ80*lac*Z ∆M15), *sup*E44, *hsd*R17, *rec*A1, *end*A1, *gyr*A96, *thi*-1, *rel*A1	[43]
S17-1	*recA*, *pro*, *hsdR*, RP4- 2Tc∷Mu Km∷Tn7 integrated into the chromosome	[46]
*C. glutamicum*		
WT	ATCC13032	ATCC
MDS0	ATCC13032 Δ*ald* Δ*fadH*	[41]
MDS1	ATCC13032 Δ*ald* Δ*fadH* Δ*rpi* (pEKEx3-*xylAB)*	This study
MDS2	ATCC13032 Δ*ald* Δ*fadH* Δ*rpe* (pEKEx3-*mdh*-*hxlAB)*	This study
MDS2T8	strain evolved from MDS2; after 8 transfers	This study
MDS2T14	strain evolved from MDS2; after 14 transfers	This study
MDS2 Δcg3104	MDS2 with deletion Δcg3104	This study
MDS2 Δcg3104 Δ*metY*	MDS2 Δcg3104 with deletion Δ*metY*	This study
MDS2 *metK*_S288N	MDS2 with SNP in *metK* resulting in amino acid exchange S288N	This study
MDS2 Δcg3104 *metK*_S288N	MDS2 with deletion Δcg3104 and SNP in *metK*, resulting in amino acid exchange S288N	This study
MDS2T14 *metK*_N288S	MDS2T14 with reversion of *metK* to the WT sequence	This study

**Table 3:** Plasmids used in this study.

Plasmid	Relevant Characteristics	Reference
pEC-XT99A	Tet^R^, *C. glutamicum*/*E. coli* shuttle vector (P_trc_ *lacI^q^*, pGA1, oriV*_E.c._*)	[47]
pEC-XT99A-*mdh*-*hxlAB*	pEC-XT99A expressing *mdh* from *B. methanolicus* and *hxlA* and *hxlB* from *B. subtilis*	This study
pEC-XT99A-*lysC*^fbr^-*ldcC*	pEC-XT99A expressing feedback-resistant *lysC* from *C. glutamicum* and *ldcC* from *E. coli*	This study
pEKEx3	Spec^R^, *C. glutamicum*/*E. coli* shuttle vector (P_tac_ *lacI^q^* pBL1, o*riV_Ec_*)	[48]
pEKEx3-*mdh*-*hxlAB*	pEKEx3, expressing *mdh* from *B. methanolicus* and *hxlA* and *hxlB* from *B. subtilis*	[20]
pEKEx3-*xylAB*	pEKEx3, expressing *xylA* from *X. campestris* and *xylB* from *C. glutamicum*	[49]
pK19*mobsacB*	Kan^R^, mobilizable *E. coli* vector mutagenesis (*oriV*, *sacB*)	[44]
pK19*mobsacB*-∆*rpi*	pK19*mobsacB* with a deletion construct *rpi*	This study
pK19*mobsacB*-∆*rpe*	pK19*mobsacB* with a deletion construct *rpe*	This study
pK19*mobsacB*-Δcg3104	pK19*mobsacB* with a deletion construct cg3104	This study
pK19*mobsacB*-*metK*_S288N	pK19*mobsacB* with an amino acid exchange *metK*_S288N from *C. glutamicum*	This study
pK19*mobsacB*-*metK*_N288S	pK19*mobsacB* with WT *metK* for reversion of amino acid exchange S288N in MDS2T14	This study
pVWEx1	Kan^R^, *C. glutamicum*/*E. coli* shuttle vector (P_tac_, *lacI^q^*)	[50]
pVWEx1-*mdh*-*hxlAB*	pVWEx1 expressing *mdh* from *B. methanolicus* and *hxlA* and *hxlB* from *B. subtilis*	This study
pVWEx1-*rpi*	pVWEx1 expressing *rpi* from *C. glutamicum*	This study

## Supplementary Materials

Supplementary materials can be found at https://www.mdpi.com/1422-0067/21/10/3617/s1. The oligonucleotides used as primers are shown in Table S1.
