# Supplementary material for: Methanol-Essential Growth of Corynebacterium glutamicum: Adaptive Laboratory Evolution Overcomes Limitation due to Methanethiol Assimilation Pathway
Source: Int J Mol Sci. 2020 May 20;21(10):3617. doi: 10.3390/ijms21103617 (PMC7279501; doi:10.3390/ijms21103617)
Supplement: Supplementary file 1 [file ijms-21-03617-s001.pdf]

Supplementary data

Methanol-essential growth of *Corynebacterium glutamicum*: Adaptive laboratory evolution overcomes limitation due to methanethiol assimilation pathway

Guido Hennig [1#], Carsten Haupka [1#], Luciana F. Brito [1,2], Christian Rückert [3], Edern Cahoreau [4], Stéphanie Heux [4], Volker F. Wendisch [1*]

Table S1. Oligonucleotides used as primers.

Oligonucleotide	Sequence
rpi-up_F	5'-TGCAGGTCGACTCTAGAGCGCACCTTTAGGGCGTATGG-3'
rpi-up_R	5'-GGGTAGGTGATTTGAATTTGTTCCAAGGTATACGCGCATGG-3'
rpi-dn_F	5'-ACAAATTCAAATCACCTACCCCGCACTGGAATCGCACCT-3'
rpi-dn-R	5'-CGACGGCCAGTGAATTCCGAGCCTTGGTGGGCAAG-3'
rpi-ko_F	5'-CTTGGCGGCGTCTACATTTC-3'
rpi-ko_R	5'-ACTACTACCCTGGCGGTAAC-3'
rpi_F	5'-ATTACGCCAAGCTTGCATGCCTGCAGAAAGGAGGCCCTTCAGATGCGCGTATACCTTGGAGC-3'
rpi_R	5'-AGTGAATTCGAGCTCGGTACCCGGGTTATTCGTTAGGAACGACAGGTGC-3'
mdh_F	5'-ATTACGCCAAGCTTGCATGCCTGCAGAAAGGAGGCCCTTCAGATGACAACAAACTTTTTCATTCCACCAG-3'
hxlB_R	5'-AGTGAATTCGAGCTCGGTACCCGGGGATCCCTATTCAAGGTTTGCGTGGTGAG-3'
pK19_F	5'-CGCCAGGGTTTTCCCAGTCACGAC-3'
pK19_R	5'-AGCGGATAACAATTTCACACAGGA-3'
exp_F	5'-GCGCCGACATCATAACGG-3'
exp_R	5'-GGCGTTTCACTTCTGAGTTCGG-3'
cg3104-up F	5’-CCTGCAGGTCGACTCTAGAGACAAAACTGAAGCGATGCCCAAAG-3’
cg3104-up R	5’-AATTGCCCTGAGCTTGTTCGCTGGTCACTTCT-3’
cg3104-dn F	5’-CGAACAAGCTCAGGGCAATTCCAGTTTCTCTAGGATCG-3’
cg3104-dn R	5’-ATTCGAGCTCGGTACCCGGGCGCCACCACCTCCGCTGTAAAAC-3’
*metK*_S288N F	5’-GCATGCCTGCAGGTCGACTCTAGAGTTGGCGCACCGACTGTCACGTCG-3’
*metK*_S288N R	5’-AGTGAATTCGAGCTCGGTACCCGGGGGGTTTTTGCCATGAATCCGAAGATACTACA-3’
cada F	5’-TTCGAGCTCGGTACCCGGGGATCCTCTAGAGAAAGGAGGCCCTTCAGGTGGCCCTGGTCGTACAG-3’
cada R	5’-AAGCTTGCATGCCTGCAGGTCGACTCTAGATTATCCCGCCATTTTTAGGACTCG-3’
gntK F	5’-ACCGTGCATGGTGGCTAGTC-3’
gntK R	5’-GCAAGGTGGATGGCATGAAG-3’
oriV F	5’- GCAAGGTGGATGGCATGAAG-3’
oriV R	5’- AGCATGTTGCGGTGCAAGTG-3’
